# Investigating Commercial Filaments for 3D Printing of Stiff and Elastic Constructs with Ligament-Like Mechanics

**DOI:** 10.3390/mi11090846

**Published:** 2020-09-11

**Authors:** Audrey A. Pitaru, Jean-Gabriel Lacombe, Megan E. Cooke, Lorne Beckman, Thomas Steffen, Michael H. Weber, Paul A. Martineau, Derek H. Rosenzweig

**Affiliations:** 1 Division of Orthopaedic Surgery, McGill University, Montreal, QC H3A 1A1, Canada; audrey.pitaru@mail.mcgill.ca (A.A.P.); jean-gabriel.lacombe@mail.mcgill.ca (J.-G.L.); megan.cooke@mail.mcgill.ca (M.E.K.); Michael.weber@hotmail.com (M.H.W.); paul.martineau@mcgill.ca (P.A.M.); 2 Department of Experimental Surgery, McGill University, Montreal, QC H3A 1A1, Canada; 3 The Orthopaedics Research Lab, McGill University, Montreal, QC H3A 1A1, Canada; lornebeckman@gmail.com (L.B.); tsteffen@orlmcgill.org (T.S.); 4 Injury, Repair and Recovery Program, Research Institute of McGill University Health Centre, Montreal, QC H3A 1A1, Canada

**Keywords:** 3D printing, scaffolds, tissue engineering, elastic, mechanical strain, ligament, polymers

## Abstract

The current gold standard technique for treatment of anterior cruciate ligament (ACL) injury is reconstruction with autograft. These treatments have a relatively high failure and re-tear rate. To overcome this, tissue engineering and additive manufacturing are being used to explore the potential of 3D scaffolds as autograft substitutes. However, mechanically optimal polymers for this have yet to be identified. Here, we use 3D printing technology and various materials with the aim of fabricating constructs better matching the mechanical properties of the native ACL. A fused deposition modeling (FDM) 3D printer was used to microfabricate dog bone-shaped specimens from six different polymers—PLA, PETG, Lay FOMM 60, NinjaFlex, NinjaFlex-SemiFlex, and FlexiFil—at three different raster angles. The tensile mechanical properties of these polymers were determined from stress–strain curves. Our results indicate that no single material came close enough to successfully match reported mechanical properties of the native ACL. However, PLA and PETG had similar ultimate tensile strengths. Lay FOMM 60 displayed a percentage strain at failure similar to reported values for native ACL. Furthermore, raster angle had a significant impact on some mechanical properties for all of the materials except for FlexiFil. We therefore conclude that while none of these materials alone is optimal for mimicking ACL mechanical properties, there may be potential for creating a 3D-printed composite constructs to match ACL mechanical properties. Further investigations involving co-printing of stiff and elastomeric materials must be explored.

## 1. Introduction

The anterior cruciate ligament (ACL) is one of the most frequently injured structures during sporting or high impact activities [1]. It is one of two cruciate ligaments in the knee and ensures passive restraint of anterior translation and rotation of the tibia with respect to the femur, in part due to its anisotropic properties [2,3,4]. The ACL is composed of a highly organized collagen matrix consisting mainly of type I and III collagen, as well as elastin, proteoglycans, glycosaminoglycans, and adhesion proteins [5,6]. The ACL has been described as a continuum of fibres [7,8,9] or as three distinct fibre bundles [10,11,12]. In all cases, it is clear that the fibres of the ACL align in the same direction, parallel to the long axis of the ligament [8,13,14,15]. The aligned collagen fibres allow the ligament to withstand sizable forces with movement [16]. ACL injuries can result in reduced functional performance but are also associated with long-term clinical conditions that include meniscal tears, cartilage lesions and an increased risk of early onset post-traumatic osteoarthritis (OA) [17]. In traumatic joint injuries, the ACL tends to fail and tear until the ends are completely separated and the recovery process is slow due to its poor healing capacity often attributed to the avascular nature of the tissue [14,18]. Additionally, reattachment of torn ends has had limited clinical success [19]. The current gold standard of treatment for ACL injury is surgical reconstruction with autograft, most commonly of the patellar or hamstring tendons. However, autograft techniques are associated with donor site-related issues such as larger incisions and anterior knee pain [18]. Other drawbacks include the amount and availability of autograft tissues and complications related to graft harvesting [20]. Further, ligament does not fully recover following viscoelastic elongation [21]. Crawford et al. [22] performed a ten-year follow up for clinical outcomes after intra-articular non-artificial ACL reconstruction where they found that the overall cumulative ACL failure rate was 11.9%. In addition, it was found that one in nine patients who undergo ACL reconstruction will have a re-rupture or clinical failure at long-term follow up.

Additive manufacturing, also known as three-dimensional (3D) printing, is a fabrication process by which 3D constructs are built in a layer-by-layer fashion using 3D computer-generated models. Additive manufacturing is open source and used for a variety of applications due to its ability to produce geometries and parts that are too complex for long-standing manufacturing processes that are subtractive in nature [23,24]. 3D printing of polymeric scaffolds can generate mechanically competent structures that can act as templates for tissue formation and regeneration [25,26]. 3D printing can also create sophisticated, porous scaffolds with complex geometry not possible with traditional manufacturing processes [27,28]. Using fused deposition modelling (FDM), many 3D printing parameters can be controlled. This enables a customizable approach to ensure that scaffold structural properties such as fibre orientation, pore size, and geometry can be optimized. A variety of biocompatible and resorbable materials have been used to generate ligament/tendon-like scaffolds such as poly-L-lactic acid, poly (urethaneurea) and polycarbonate poly (urethaneurea) [29]. However, there are very few studies into the use of 3D manufacturing and FDM for fabrication of scaffolds mimicking the biomechanical properties of the ACL.

To address shortcomings associated with surgical intervention and the poor healing of the ACL, researchers have explored tissue engineering (TE) and regenerative medicine strategies which aim to combine cells, scaffolds, and biologically active molecules [30]. 3D-printed scaffolds can be seeded with cells and then implanted into the injured site to allow for growth or regeneration of the tissue [28,31,32]. One concept is to use bioreactor-matured tissue engineered scaffolds to overcome the current limitations associated with surgical reconstruction. Furthermore, cell-seeded scaffolds form autografts that can overcome issues with supply shortage [33,34]. The implementation of tissue engineering harmonizes additive manufacturing and cell therapy to obtain state of the art tissue repair. However, it remains unclear as to whether 3D-printed polymers can be manufactured to match the biomechanical properties of the ACL.

In this study, low-cost FDM 3D printing was used to generate scaffolds from multiple materials with differing raster angles, and their mechanical properties were determined using tensile testing. The materials tested include polylactic acid (PLA), polyethylene terephthalate glycol (PETG), Lay FOMM 60, NinjaFlex, NinjaFlex-SemiFlex, and FlexiFil. PLA and PETG were selected due to their high ultimate tensile strengths (UTS). All other filaments were selected for their flexible nature. Although data on the stress properties are available for most of these materials in bulk, the effect of printing raster angle on their mechanical properties is unknown. Therefore, this study aims to fill this gap by testing not only different polymers, but also three differing raster angles to identify optimal combinations to match the mechanical strength of the ACL.

## 2. Materials and Methods

### 2.1. 3D Printing of Tensile Specimens

**Figure 1:**
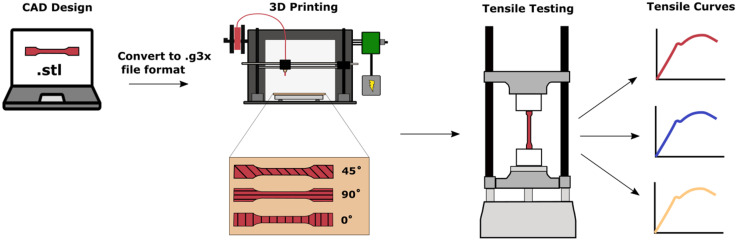
Schematic of overall procedure for 3D printing and mechanical testing of tensile specimens.

An overview of the 3D printing and tensile testing process is shown in Figure 1. 3D printing was carried out with two FDM printers, the FlashForge Creator Pro (Flashforge; Los Angeles, CA, USA) and the Monoprice MP Select Mini v2 (Monoprice, Inc; Brea, CA, USA). Design and conversion processes were similar for both printers. SOLIDWORKS 2015 (Dassault Systèmes, SolidWorks Corporation, Waltham, MA, USA) was used to design a dog bone shape for tensile testing to induce fracture in the gauge section of the specimen. The CAD file was saved as a stereolithography (STL) file and sliced using Ultimaker Cura 4.3.0 (Ultimaker B.V.; Utrecht, Netherlands) software to obtain the G code for the Monoprice printer, and a x3g file extension for the FlashForge printer. The nozzle diameter for both printers was 0.3 mm, the line width was 0.4 mm, and printing was performed indoors in a temperature-controlled environment. The infill for all specimens was 100%, with no distance between printed lines.

For each material, a total of 18 tensile specimens were produced. There were three different printing directions for each material, 0°, 90°, and 45° (see Figure 1). The 0° raster angle was defined as horizontal filling along the width of the specimen, whereas the 90° raster angle was printed longitudinally and the 45° raster angle diagonally. For the 45° specimens, the angle alternated between printed layers.

### 2.2. Filaments

The materials tested were: white PLA (MakerGeeks; Springfield, MO, USA); black PETG; (SUNLU; Commerce, CA, USA); white Lay FOMM 60 (MatterHackers; Burbank, CA, USA); blush NinjaFlex (Fenner Drives; Manheim, PA, USA); red NinjaFlex-SemiFlex (Fenner Drives; Manheim, PA, USA); and blue FlexiFil (Formfutura; Nijmegen, Netherlands). PLA and PETG were selected due to their high UTS, since the ACL is a load-bearing band in the knee. NinjaFlex is a thermoplastic polyurethane composition that allows for 660% elongation. NinjaFlex-SemiFlex is made of the same polymers, but is formulated to be slightly more rigid, to allow for an increased UTS. FlexiFil is a rubberized thermoplastic co-polyester. These three filaments were selected due to their flexible nature, in an attempt to mimic the viscoelastic nature of the native ACL. Lay FOMM 60 is a highly porous material composed of a flexible thermoplastic polyurethane (TPU) and polyvinyl alcohol (PVA) blend. The PVA component is water-soluble and can be washed out to create flexible, nanoporous, sponge-like structures following printing [35]. The manufacturer-reported mechanical properties for the used materials can be found in Table 1. Lay FOMM 60 did not have any reported mechanical properties from the manufacturer.

The Lay FOMM 60 filament was heated in an oven for three hours at 80 °C before printing to remove all moisture. The printed specimens were rinsed for three days in double distilled water to wash out most of the rigid PVA, replacing the water each day.

The printing parameters used for each material can be found in Table 2. For PLA, layer height was set to 0.2 mm, flow rate was set to 100%, retraction was enabled, and the printing speed was offset between 65% and 90% based on the appearance of the tensile specimen. Similar settings were applied to PETG but a skirt and raft were used to enhance build plate adhesion, and the print speed was set to 100%. As for NinjaFlex-SemiFlex, referred to here as SemiFlex, initial layer height was set to 0.3 mm, flow rate was set to 102% offset, retraction and combing were enabled, and the printing speed was set between 55% and 65% offset. The same settings were applied to FlexiFil, but the print speed was set to 100%. The NinjaFlex filament had an initial layer height of 0.3 mm, a skirt was used, the flow rate was set to 110%, and retraction was disabled. Finally, Lay FOMM 60 was printed with initial layer height of 0.2 mm using a skirt, the flow rate set to 100%, retraction enabled, and a printing speed set between 40% and 95% offset.

### 2.3. Light Microscopy

**Figure 2:**
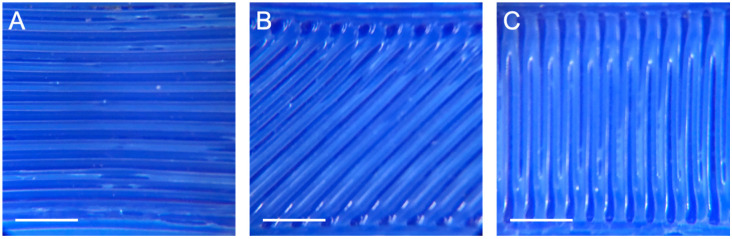
Light microscopy images of FlexiFil printed with raster angles of (**A**) 90°, (**B**) 45° and (**C**) 0°. Scale bar = 2.5 mm.

The microscopic images of the printed constructs were captured with a Leica MS5 stereomicroscope, using the 1.6 magnification lens adapter for iPhone. Sample light microscopy images of the three raster angles are shown in Figure 2.

### 2.4. Mechanical Testing

Tensile testing was performed using the Mini-Bionix 858 (MTS; 14,000 Technology Dr. Eden Prairie, MN, USA). All specimens were tested with the TestStar II (MTS; 14,000 Technology Dr. Eden Prairie, MN, USA) software. A large, built-in MTS, Model #662.20D-03 load cell was used in the calibrated 2000 N range. A smaller Model LCCD-100 (Omegadyne; 800 Connecticut Ave. Suite 5N01, Norwalk, CT 08654, USA) load cell with a capacity of 444 N was used for the Lay FOMM 60 specimens. The force and displacement data were recorded at 10 Hz. The displacement was set to a maximum of 95 mm. The test speed was 0.3 mm/second corresponding to 1% strain/second based on a nominal 30 mm gauge length. All tensile tests were performed at ambient room temperature. Lay FOMM 60 was stored in water until three hours before testing. The gauge, width, and thickness of the gauge were measured using a caliper.

### 2.5. Tensile Analysis

All force and displacement data were converted to stress and strain data using the specimens’ cross-sectional areas and initial gauge lengths. Stress and strain were calculated by Equations (1) and (2), respectively.

(1) $$Stress\;\left(Pa\right)=\frac{Force\;\left(N\right)}{Area\;\left(mm^{2}\right)}$$

(2) $$Strain\;\left(\%\right)=\frac{Displacement\;\left(mm\right)}{Original\;length\;\left(mm\right)}$$

To ensure the replicability of analyses, force and displacement data were zeroed at the same points for all materials prior to conversion to stress and strain data. The stress–strain curves of PLA and PETG were used to calculate the Young’s modulus, UTS and percent strain at failure. The Young’s modulus for PLA and PETG was calculated as the ratio of tensile stress to tensile strain in the linear portion of each stress–strain curve (Equation (3)). UTS and percent strain at failure were observed directly from the stress–strain curve.

(3) $$Young’s\;and\;Apparent\;Modulus=\;\frac{Stress}{Strain}$$

Conventional UTS, percent strain at failure and yield strength values were not used to characterize the flexible materials due to atypical failures (occurring past 100% strain). An apparent Young’s modulus and atypical yield point were defined to assess the change in tensile elongation behavior as the flexible specimens are stretched past their elastic limit. The apparent Young’s modulus used to describe flexible materials was obtained by determining the ratio of tensile stress to tensile strain (Equation (3)) in a restricted region of interest between strains of 0 and 5%. This region of interest limits the analysis to a semi-linear curve that approximates the stiffness of flexible polymers and acts as a more accurate benchmark for elasticity analysis since a conventional Young’s modulus cannot be used. The flexible yield point was defined as the stress and strain values at which a 2% offset line parallel to the semi-elastic slope of the stress–strain curve intersects the non-linear portion of the curve. The flexible yield point was used to approximate the point at which flexible materials start to experience plastic deformation. The stress–strain curves of flexible materials were used to determine additional properties of stress at 5% strain and stress at 20% strain.

### 2.6. Verification of Gauge Displacement

Due to the elastic nature of a number of tested materials, the gauge elongation was verified to ensure that the grip section elongation was negligible. The open-source Tracker video analysis and modeling tool was used to assess the movement of two points at opposite ends of the gauge section. Videos were recorded using an iPhone camera and uploaded to the software for analysis. Three independent Lay FOMM 60 tensile tests for each raster angle were performed.

### 2.7. Statistical Analyses

At least five independent tensile tests for each material and each raster angle were used to conduct the statistical tests, with the exceptions of PLA 0° and PETG 0° raster angles which each had 3 independent tests. Statistical analyses of stiff and flexible materials were separated into 2 groups due to their mechanics; comparisons were made within and not across groups. Statistical analyses of stiff materials, PLA and PETG, were performed comparing UTS, Young’s modulus, and percent strain at failure within raster angles using unpaired t tests. Statistical analyses for flexible materials for each raster angle were also performed comparing their apparent modulus and the stress at 5% and 20% strain using an ordinary one-way analysis of variance (ANOVA). Tukey HSD post-hoc tests were performed for comparisons between materials with the same raster angle, as well as between the three raster angles for each material. *p* < 0.05 was considered statistically significant. Analyses were performed using GraphPad Prism 6.0 (GraphPad Software, La Jolla, CA, USA).

## 3. Results

### Tensile Properties

PETG and PLA showed traditional deformation profiles, while the other materials showed more elastomeric behavior. For this reason, they were split into two groups for analysis: stiff (PLA and PETG) or flexible (Lay FOMM, SemiFlex, NinjaFlex and FlexiFil). The specimens were designed to fail in the gauge region. However, four PLA-0° specimens failed above the gauge region. This could be due to a defect in the specimen; consequently, all four specimens were excluded from the data. Most of the flexible materials did not reach failure as displacement was limited to 90 mm by the Mini-Bionix testing machine. The only flexible materials that failed were FlexiFil and Lay FOMM 60, both at 0° and 45° raster angles. For Lay FOMM 60-0° samples, the outline of the specimen did not adhere well to the infill, which caused the shape of the specimen to warp. To overcome this, the excess filament of the outline was trimmed off using scissors prior to testing.

**Figure 3:**
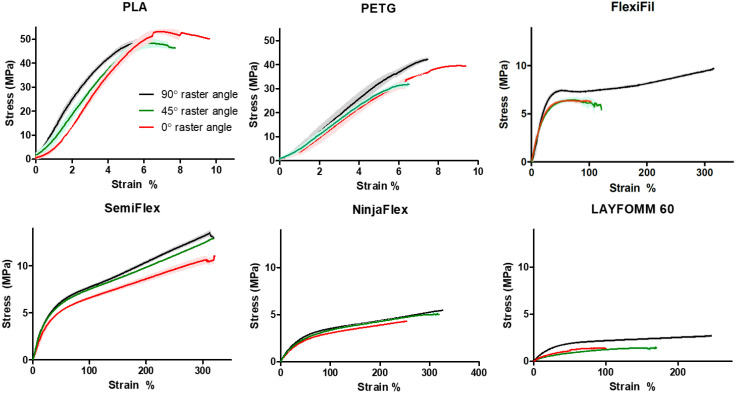
Stress–strain curves for all materials (*n* = 3) at each raster angle. Shaded zones around plot lines indicate the mean ± SD. In some samples, error bars (SD) are too small to be displayed.

Stress–strain curves for all materials are shown in Figure 3. Individual curves were used to determine the UTS, Young’s modulus, and the strain percentage at failure for the stiff specimens, and apparent modulus and the stress at 5% and 20% strain for the flexible specimens. For both PLA and PETG, clear regions of linear behavior are seen in the low strain percentage regions (at all raster angles). When considering the flexible materials, the stress–strain behavior of these polymers does not follow the typical tensile curve with an elastic region followed by a plastic region and in most cases, such as FlexiFil-90°, is more akin to elastomeric deformation. The linear portion commonly seen in low-strain regions was not distinct for NinjaFlex or Lay FOMM 60.

**Figure 4:**
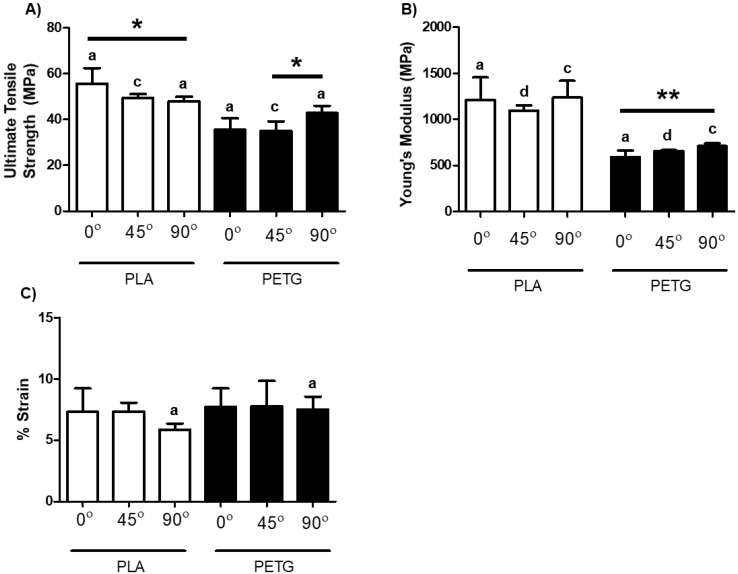
Mechanical properties of PLA and PETG at three differing raster angles: 0°, 45°, and 90°. (**A**) UTS; (**B**) Young’s modulus; (**C**) percent strain at failure. The letter denotation indicates a significant difference exists between PLA and PETG within the same raster angle. ^a^ Significant difference of *p* < 0.05. ^b^ Significant difference of *p* < 0.01. ^c^ Significant difference of *p* < 0.001. ^d^ Significant difference of *p* < 0.0001. * *p* < 0.05. ** *p* < 0.01. Data shown represent the mean ± SD. ^a–d^ indicates significant differences comparing the same raster angle between different materials, while * indicates significant differences between raster angles of the same material.

The calculated UTS, Young’s Modulus and percentage strain at failure for stiff materials are shown in Figure 4; corresponding results of one-way ANOVA of the effect of raster angle are shown in Table 3. In the case of PETG, raster angle had a significant effect on UTS and Young’s modulus as shown in Figure 4A,B, respectively. The strain at failure for PETG was not significantly different. As for PLA specimens, Table 3 shows that there were statistically significant differences for UTS and strain at failure, but no significant difference for the Young’s modulus. PLA was unique in that the trend of UTS and percentage strain at failure was opposite to all other materials, with 0° having significantly higher values than 90°. In terms of the comparison between materials, Figure 4A,B demonstrate statistically significant differences for UTS and Young’s modulus between PLA and PETG specimens at each raster angle. As for percent strain at failure (Figure 4C), only at 90° were PLA and PETG significantly different (*p* = 0.0317).

**Figure 5:**
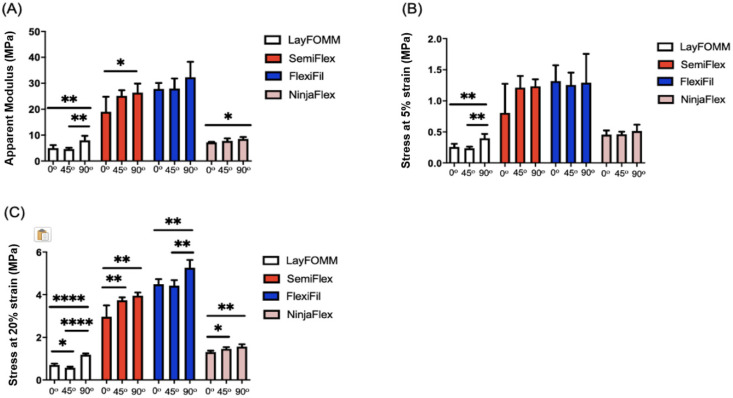
Mechanical properties of the Lay FOMM 60, SemiFlex, FlexiFil, and NinjaFlex materials for three raster angles: 0°, 45°, and 90°. The mechanical properties displayed are: (**A**) apparent modulus; (**B**) stress at 5% strain; (**C**) stress at 20% strain. * *p* < 0.05, ** *p* < 0.01, **** *p* < 0.0001. Data shown represent the mean ± SD.

Figure 5 shows calculated apparent modulus (Figure 5A), stress at 5% strain (Figure 5B) and stress at 20% (Figure 5C) strain for flexible materials; corresponding results of one-way ANOVA of the effect of raster angle are shown in Table 4. Raster angle caused a statistically significant difference in apparent moduli of Lay FOMM 60, SemiFlex, and NinjaFlex (Table 4). Further, apparent moduli were significantly different between raster angles of 0° and 90° in these three materials. There were no significant differences in apparent moduli either between angles or within the three angles for FlexiFil. There are statistically significant differences between raster angles in terms of stress at 5% strain (Figure 5B) only for Lay FOMM 60. A difference exists between both 0° and 90° (*p* = 0.0031), and 45° and 90° (*p* = 0.0011) raster angles. No significant differences were found for SemiFlex, FlexiFil and NinjaFlex. The stresses at 20% strain were statistically significant within all flexible materials, as can be seen in Figure 5C. Lay FOMM 60 was the only flexible material in which apparent moduli and the stress at 5% and 20% strain were significantly different within the material for all three raster angles (Table 4).

A comparison of the significance values for the apparent modulus and the stress at 5%, 20%, 50% and 100% strain for the flexible specimens for the 0°, 45°, and 90°raster angle can be found in the supplementary data (Tables S1–S3). Significant differences were apparent for most of the material comparisons at 5% strain for raster angle 0° (Table S1). At strains of 20% and 50% for the same raster angle, significant differences were found for all materials. Data for stress at 100% strain for Lay FOMM are not available since the specimens failed before reaching the latter strain. At raster angles of 45° and 90°, most comparisons were significantly different. Overall, Lay FOMM 60 and NinjaFlex were the only materials that were consistently not statistically different.

**Figure 6:**
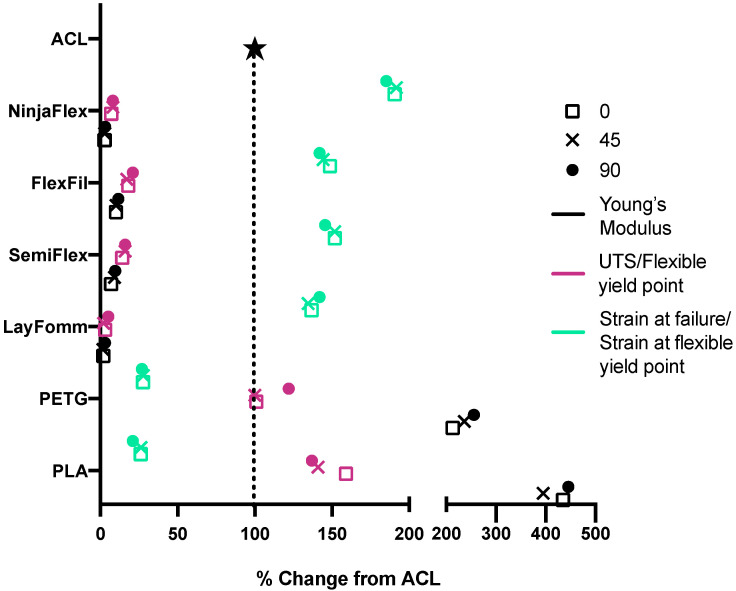
Percentage change of values compared to published native ACL mechanical properties. Raster angles are represented as 0°—square, 45°—cross, and 90°—circle. Young’s (stiff) or apparent (flexible) modulus values are shown in black, UTS (stiff) or flexible yield point (flexible) are pink and strain at either failure (stiff) or flexible yield point (flexible) are green. Mean values are plotted.

The experimental findings were then compared to previously published values of ACL mechanical properties, using a Young’s modulus value of 278 MPa [41], UTS of 35 MPa [41], and strain at failure of 28% [42]. More specifically, these data were compared to the mechanical properties of all six materials with the ACL data normalized to 100% (shown graphically in Figure 6, mean values are presented in Table 5). The Young’s modulus values of PLA and PETG were around 2.5 and 4-fold greater than the ACL. As for UTS, the PETG specimens at 0° and 45° were very close in value to the UTS of the ACL, whereas the PLA specimens were slightly higher. The stiff materials did, however, have strain at failure values that were much lower the ACL. Opposite trends were seen for the flexible materials. The apparent moduli and flexible yield point were much lower than the native ACL’s Young’s modulus and the strain at flexible yield was higher than that of the ACL for all flexible materials at all raster angles. FlexiFil, SemiFlex and Lay FOMM had strain at flexible yield values around 1.5-fold higher than the native ACL, but NinjaFlex was 2-fold higher.

## 4. Discussion

ACL tears are very common; the current standards for surgical treatment are not as strong as the original ligament and have relatively high failure and re-tear rates. More effective ligament reconstruction strategies are therefore necessary. The fabrication of scaffolds for tendon and ligament tissue engineering has utilized numerous synthetic biomaterials, such as polycaprolactone, polyglycolic acid, poly(lactic-co-glycolic acid), poly-L-lactide, and polyurethane urea, as well as other techniques: electrospinning, knitting, melt extrusion-based 3D-bioplotting, and 3D braiding [13,30]. The technique of FDM in particular has been used to print different polymers to determine their tensile properties [43]. However, no studies to our knowledge have investigated raster angle in FDM printing to optimize biomaterial mechanics for ACL reconstruction. The strategy used for 3D printing in this study was a good fit. We produced tensile specimens that had uniform surface appearance, despite the use of different materials with varying properties. Tensile testing was then performed to determine the effect of changing raster angle on their appropriateness for use as mechanically functional ligament replacements.

Values for the mechanical properties of the ACL are inconsistent in the literature. It is difficult to assess the cross-sectional area of the ACL and to define an appropriate gauge length, which are needed to calculate the stress and strain. Thus, tensile properties of the ACL are often described in terms of force and elongation [44]. However, these properties are influenced by the specific geometry of each construct, which means they cannot be compared to anything other than ACL data. For the purposes of this study, stress and strain were chosen as the properties to be calculated since they allow for the possibility of comparison between the ACL and other materials, despite the fact that limited data on stress and strain for the ACL are available. It is also important to note the rationale behind selecting certain values for the mechanical properties. There is no literature on 3D printing of elastomers to mimic ligaments and therefore no data for direct comparison. The stress at 5% strain was selected as peak strains produced during activities of daily living have been found to be close to 4% for the ACL [45]. The maximum strain for the ACL has been reported between 14.0% and 14.4% [46] in some studies, and close to 19.1% in others [47]. A 20% strain was selected as it is above the values reported. It is important to note that stress at 100% and 300% strains is common for stress–strain curve analysis of elastomers. Despite being of limited relevance for the study of the ACL, stresses at 100% strain were still included to further characterize the materials which may be applicable in other, more flexible ligaments or tissues.

3D printing is an established technique in a number of industries, and it is interesting to explore the feasibility of 3D-printed thermoplastics for ACL substitutes. PETG showed UTS values that were very close to those of the ACL (37.77 vs. 35 MPa). The Young’s modulus was higher than that of the ACL (652.87 vs. 278 MPa) and the strain at failure was lower (7.68 vs. 28%). These tensile values do not allow for a satisfactory factor of safety and therefore signifies that PETG scaffolds printed using the stated parameters may not be viable mechanical substitutes for the ACL despite presenting similar tensile properties. PLA constructs presented Young’s modulus (1181 MPa) and UTS (51.04 MPa) values which were above those of the ACL. However, its strain at failure is lower than ACL values found in literature (6.835 vs. 28%). The apparent moduli, stress at 5% and 20% strains, and the flexible yield point of all flexible materials was significantly lower than that of the native tissue. Only the strain at flexible yield values for all flexible materials were larger than the ACL’s strain at failure. Because the Young’s modulus and UTS of stiff materials are higher than that of the ACL, it may be possible to co-print them with a flexible material in order to increase the strain at failure and produce a composite scaffold with tensile properties very close to that of the ACL. Lay FOMM 60 is a good candidate for co-printing with a stiff material. In the removal of PVA with washing in water, micropores are revealed in the Lay FOMM surface. Numerous studies have shown the importance of porosity in scaffolds for cell attachment and proliferation [28,48] and the use of Lay FOMM 60 with PLA or PETG may enhance such properties. Our group has already reported that Lay FOMM 60 shows good cell viability in vitro and can deliver chemotherapeutics [35,49]. Lay FOMM’s ability to deliver small molecules may enable the delivery of growth factors or hormones to enhance proliferation and matrix formation of ligament fibroblasts.

The impact of raster angle on the tensile properties was also investigated. It has been shown that aligned fibres result in higher tensile properties in scaffolds [50], and that increased fibre alignment leads to increased tissue stiffness [51,52]. Fibres must be printed in all orientations to replicate the anisotropic properties of the native ACL. A strong decline in tensile properties between raster angles may affect the mechanical stability of the graft in tension and cause tensile failure. It is important to consider the impact of raster angle since the graft needs to perform in many tensile planes at once. Additionally, combining the flexible and stiff materials may better mimic the anisotropic properties of the ACL, which has not been accomplished thus far to our knowledge. Raster angle had a significant impact on UTS and Young’s modulus of the PETG specimens and on the UTS and strain at failure of the PLA specimens. This indicates that the tensile properties of PETG and PLA constructs fabricated using the stated parameters can be altered by their fibre orientation. Such alterations may be beneficial in replicating the ACL’s anisotropic behavior. Flexible materials were all significantly impacted by the raster angle, except for FlexiFil. The raster angle had a significant effect on all mechanical properties for Lay FOMM 60 and all mechanical properties except for 5% strain for NinjaFlex and SemiFlex. As for FlexiFil, the only mechanical properties that were impacted by the raster angle were the strains at 20%, 50% and 100%, whereas the apparent modulus and 5% strain were not statistically significant. Apparent modulus and 5% strain are very important parameters regarding the mechanical properties of the ACL, so changing raster angle can be considered not important for FlexiFil. In conclusion, in terms of raster angle, Lay FOMM 60, SemiFlex, and NinjaFlex may be used to replicate the anisotropic properties of the ACL and may be more appropriate flexible co-printing candidates.

Use of FDM for fabrication of ACL scaffolds presents limitations. First, materials that possess appropriate properties for FDM such as viscoelasticity, thermoplasticity, and melting/solidification are limited. Further, the use of commercially sourced materials means that their exact composition is often proprietary (as in this study). Scaffold design is also restricted since the viscosity of the molten polymers only allow for the fabrication of structures with a bottom-up design approach. A limitation of the current study was the lack of testing to ensure that there was not under-extrusion of the interior of the specimen. Future studies should include precision weighing of the specimens in order to prevent this issue. If under-extrusion is found, the specimens should then be reprinted since mechanical stability is imperative in the ACL and previous studies have indicated a correlation between polymer weight and mechanical properties [53]. According to Perego et al. [54], the tensile strength was affected by the molecular weight of the specimen, whereby a larger molecular weight resulted in a higher tensile strength. Furthermore, Wittbrodt and Pearce [55] found that colouring agents altered the percent crystallinity, which had an impact on tensile strength. Wimpenny et al. [56], however, stated that colour of the filament has little effect on the tensile strength of FDM constructs. As no consensus has been reached in the literature, the colour of the filament was not explored in this study. It is known that the crystallinity impacts the tensile strength [55,57], therefore future studies should evaluate percent crystallinity of the material, weigh the specimens, and use uncoloured, natural filament to prevent confounding contributions of colourants and their sources. Another source of uncertainty is torsional forces. Although we investigated the tensile properties of polymeric materials, the response to torsional stresses and strains is unknown. It is believed that the raster angle of 3D-printed constructs may have an impact on torsional stress distribution within the material. Due to the ACL's multi-axial range of motion, it is important to also investigate these properties to ensure the mechanical accuracy of the scaffolds. Such torsional stresses could not be investigated in this study but should be considered in future investigations. Additionally, the stress–strain curves shown in this study assume a fixed cross-sectional area, which is not accurate for elastomeric specimens during elongation.

The ACL mechanical properties were selected from specific publications as a point of reference [41,42]. Nevertheless, there are other studies that show different values than what we reported. Unfortunately, there is no consensus in the literature. While the ACL is certainly one of the most significant and well described ligaments, many other ligaments are critical to joint stability throughout the human body. Moreover, surgery for ligament reconstruction is common and standard of care in hand surgery, foot and ankle surgery, orthopedic trauma surgery and even spine surgery [58,59,60]. In general, ligaments of joints smaller than the knee are reconstructed using Ethicon, fibre wire, Ethibond sutures or mersilene tape. Although our 3D-printed materials were not fit for the high biomechanical demands of the knee, we believe the characteristics of these biomaterials, such as freedom of printable complex geometries, potential resorptive capacities and biomechanical strength, may prove to be a dynamic and promising alternative in many other smaller and less biomechanically demanding ligament reconstruction scenarios.

In this study, we determined the effect of raster angle on the mechanical properties of a number of stiff and flexible materials as candidates for mechanically functional ACL scaffolds. Whilst there was no single material that matched literature values for the native ACL, PLA and PETG had comparable ultimate tensile strengths and Lay FOMM 60 had the closest percentage strain at failure. A combination of PLA/PETG and Lay FOMM 60 to co-print biomimetic ACL constructs should be attempted in future studies. The mechanical properties of PLA and PETG were altered by the raster angle, which suggests that they may be used to replicate the anisotropic properties of the ACL. Co-printing with Lay FOMM 60 may increase the construct’s strain at failure and yield a more functional structure. To overcome the described shortcomings, a robust cadaveric study of the ACL should be implemented. Future studies into co-printing of these materials into composite structures and introducing porosity will hopefully enable the production of a more mechanically appropriate scaffold. Then, a comparison of several samples of cadaveric ACL to the real values of co-printed constructs should be performed. In addition, in-depth in vitro and in vivo studies with the use of scaffolds should be conducted once appropriate materials are identified with optimal mechanics and favourable biocompatibility.

## Figures and Tables

**Table 1:** Reported tensile values for raw filaments.

Material	Yield Tensile Strength (MPa)	Ultimate Tensile Strength (MPa)	Tensile Modulus (MPa)	Manufacturer Datasheet
NinjaFlex	4	26	12	[36]
SemiFlex	9	43	25	[37]
FlexiFil		24	95	[38]
PLA	35.9	26.4	2300	[39]
PETG	53		2100	[40]

**Table 2:** Printing parameters of all materials.

Material	Nozzle Temperature (°C)	Bed Temperature (°C)	Initial Layer Speed (mm/s)	Speed (mm/s)
PLA	205	70	17.5	35
PETG	240	80	17.5	35
SemiFlex	215	50	15	30
FlexiFil	215	55	15	30
NinjaFlex	230–240	55	10	15
Lay FOMM 60	220–225	50	15	25

**Table 3:** Results of one-way ANOVA comparing raster angles on mechanical properties of PLA and PETG.

Material	Effect of Raster Angle on Mechanical Properties		
	UTS	Young’s Modulus	Strain at Failure
PLA	0.0300	0.6114	0.0280
PETG	0.0232	0.0053	0.9606

**Table 4:** Results of one-way ANOVA comparing effects of raster angle on mechanical properties of flexible materials.

Material	Effect of Raster Angle on Mechanical Properties				
	Apparent Modulus	5% Strain	20% Strain	50% Strain	100% Strain
Lay FOMM 60	0.0018	0.0008	<0.0001	0.0193	N/A
SemiFlex	0.0323	0.0746	0.0012	0.0006	0.0003
FlexiFil	0.2166	0.8273	0.0012	0.0119	0.0471
NinjaFlex	0.0489	0.4141	0.0028	0.0019	<0.0001

**Table 5:** Mechanical properties of all specimens and ACL literature values.

Material		Mechanical Properties		
	Raster Angle (°)	Young’s Modulus (MPa)	UTS/Flexible Yield Point (MPa)	Strain at Failure/Strain at Flexible Yield (%)
PLA	0	1208	55.72	7.301
	45	1098	49.43	7.347
	90	1238	47.98	5.857
PETG	0	591.6	35.50	7.731
	45	656.3	34.96	7.786
	90	710.7	42.85	7.509
Lay FOMM 60	0	5.040	1.017	38.27
	45	4.622	0.8152	37.69
	90	7.992	1.711	39.76
SemiFlex	0	18.98	4.822	42.53
	45	25.16	5.605	42.47
	90	26.39	5.687	40.75
FlexiFil	0	27.82	6.129	41.63
	45	27.96	5.992	40.39
	90	32.30	7.189	39.75
NinjaFlex	0	7.244	2.404	53.38
	45	7.768	2.650	53.70
	90	8.505	2.797	51.85
ACL	-	278 [40]	35 [40]	28 [41]

## Supplementary Materials

The following are available online at https://www.mdpi.com/2072-666X/11/9/846/s1, Table S1: Results of one-way ANOVA Tukey’s multiple comparison test comparing the mechanical properties of flexible Significance values of apparent modulus, 5% strain, and 20% strain for flexible specimens having a raster angle of 0°, Table S2: Results of one-way ANOVA Tukey’s multiple comparison test comparing the mechanical properties of flexible specimens with a raster angle of 45°, Table S3: Results of one-way ANOVA Tukey’s multiple comparison test comparing the mechanical properties of flexible specimens with a raster angle of 90°.
