# Supplementary material for: Investigating Commercial Filaments for 3D Printing of Stiff and Elastic Constructs with Ligament-Like Mechanics
Source: Micromachines (Basel). 2020 Sep 11;11(9):846. doi: 10.3390/mi11090846 (PMC7570386; doi:10.3390/mi11090846)
Supplement: Supplementary file 1 [file micromachines-11-00846-s001.pdf]

micromachines

Supplementary Materials: 3D Printing to microfabricate stiff and elastic scaffolds that mimic ligament tissue

Supplementary table 1. Results of one-way ANOVA Tukey's multiple comparison test comparing the mechanical properties of flexible specimens with a **raster angle of 0°**.

Material comparison	Significance Values of Mechanical Properties				
	Apparent modulus	5% strain	20% strain	50% strain	100% strain
LayFOMM vs. SemiFlex	<0.0001	0.0231	<0.0001	<0.0001	N/A
LayFOMM vs. FlexiFil	<0.0001	<0.0001	<0.0001	<0.0001	N/A
LayFOMM vs. NinjaFlex	0.6971	0.6583	0.0261	0.0026	N/A
SemiFlex vs. FlexiFil	0.0023	0.0378	<0.0001	0.0124	0.0938
SemiFlex vs. NinjaFlex	0.0001	0.2040	<0.0001	<0.0001	<0.0001
FlexiFil vs. NinjaFlex	<0.0001	0.0006	<0.0001	<0.0001	<0.0001

Supplementary table 2. Results of one-way ANOVA Tukey's multiple comparison test comparing the mechanical properties of flexible specimens with a **raster angle of 45°**.

Material comparison	Significance Values of Mechanical Properties				
	Apparent modulus	5% strain	20% strain	50% strain	100% strain
LayFOMM vs. SemiFlex	<0.0001	<0.0001	<0.0001	<0.0001	N/A
LayFOMM vs. FlexiFil	<0.0001	<0.0001	<0.0001	<0.0001	N/A
LayFOMM vs. NinjaFlex	0.1727	0.0876	<0.0001	0.0006	N/A
SemiFlex vs. FlexiFil	0.2542	0.9599	<0.0001	0.1197	0.0429
SemiFlex vs. NinjaFlex	<0.0001	<0.0001	<0.0001	<0.0001	<0.0001
FlexiFil vs. NinjaFlex	<0.0001	<0.0001	<0.0001	<0.0001	<0.0001

Supplementary table 3. Results of one-way ANOVA Tukey's multiple comparison test comparing the mechanical properties of flexible specimens with a **raster angle of 90°**.

Material comparison	Significance Values of Mechanical Properties				
	Apparent modulus	5% strain	20% strain	50% strain	100% strain
LayFOMM vs. SemiFlex	<0.0001	0.0003	<0.0001	<0.0001	N/A
LayFOMM vs. FlexiFil	<0.0001	0.0002	<0.0001	<0.0001	N/A
LayFOMM vs. NinjaFlex	0.9958	0.8740	0.0484	0.0003	N/A
SemiFlex vs. FlexiFil	0.0817	0.9813	<0.0001	<0.0001	0.0115
SemiFlex vs. NinjaFlex	<0.0001	0.0015	<0.0001	<0.0001	<0.0001
FlexiFil vs. NinjaFlex	<0.001	0.0007	<0.0001	<0.0001	<0.0001
